# Test designs and modeling under the general nominal diagnosis model framework

**DOI:** 10.1371/journal.pone.0180016

**Published:** 2017-06-23

**Authors:** Jinsong Chen, Hui Zhou

**Affiliations:** Department of Psychology, Sun Yat-Sen University, Guangzhou, Guangdong, China; Universita degli Studi del Piemonte Orientale Amedeo Avogadro, ITALY

## Abstract

Most psychological questionnaires face issues of response bias in respondent-reported scales, inadequacy for criterion-reference testing, or difficulty in estimating a large number of latent traits. Situational tests together with the general nominal diagnosis model framework provide a viable alternative to alleviate these concerns. Under this framework, there are different ways to design situationally nominal items, which can offer more flexibility for test development. Any response bias remaining with respondent-reported questionnaires may be addressed with appropriate test designs. The saturated model subsumes different reduced forms that can help inform whether the test is designed as expected. Two simulation studies are presented to demonstrate the effectiveness of the models and designs.

## Introduction

Most existing measures of behavioral or psychological constructs for typical performance (e.g., personality, emotion, temperament, and attitude) have some drawbacks. First, many are respondent-reported in a rating or Likert scale, making them vulnerable to response biases such as extreme or moderate responding, acquiescence bias, halo/horn effect, or social desirability [1, 2]. Moreover, use of the measures relies on the assumption that the category labels are interpreted similarly across respondents and traits, which is unrealistic [3]. Second, the measures are norm-referenced on the basis of classical test theory or item response theory, making them more appropriate for interindividual comparisons with a relative standing. In practice, however, the measures are more often used to inform the absolute standing of the respondents or to make a diagnosis. For instance, parents would rather learn if their children’s temperament is calm, active, and focused than if it rates at the top 30% of their peers. In these cases, the test scores have to be transformed with post hoc cutoff scores, raising validity concerns. Third, it is not uncommon in practice to encounter constructs with a large number of related traits or attributes. It can be challenging to estimate a large number of continuous latent variables with categorical responses. Moreover, all items are assumed to be single-stimulus or unidimensional, but some traits can be difficult to separate and measure individually. For instance, there can be up to 15 traits in a child’s temperament [4], and some pairs of them (e.g., activity level vs. impulsivity, attentional focusing vs. inhibitory control) are highly connected and difficult to measure individually.

Although there are different ways to address each concern separately, this research explores the feasibility of addressing the issues jointly through the cognitive diagnosis model (CDM) framework and situational formats such as the situational judgment test. Lievens, Peeters, and Schollaert [5] and McDaniel and Whetzel [6] have described how situational judgment tests have been used in industrial-organizational psychology applications, such as personnel selection. Situational formats have also been applied in areas such as child development [7–9]. By presenting realistic, hypothetical scenarios with choices of nominal responses, situational formats can be less vulnerable to the response biases that often occur on respondent-reported questionnaires. Moreover, it is possible to create a large number of situation-specific items that are substantively different (e.g., for computerized adaptive testing or longitudinal studies), which is not straightforward to do in a respondent-reported format. In addition, situational formats are multidimensional [6], a characteristic well suited to CDMs.

CDMs are psychometric models designed to assess the strengths and weaknesses of examinees across a set of attributes. CDM-based measurement can provide finer-grained and domain-specific diagnostic information that can be used for different testing purposes. By discretizing the usually continuous latent space, CDMs can accommodate a large number of latent dimensions in various forms, which would be challenging to do otherwise. In addition, CDMs in saturated form are versatile and flexible to address the complexity of within-item dimensionality, whether compensatory or conjunctive. More importantly, by incorporating substantive knowledge through a Q-matrix [10], CDM-based measurement is criterion-referenced, capable of providing the absolute standing of examinees. By combining CDMs with situational formats and nominal responses, it should be possible to theoretically address the measurement concerns for typical performance.

Recently, considerable developments have appeared in the CDM literature. Among others, these have included highly constrained models like the *deterministic inputs*, *noisy "and" gate* (DINA) model [11] and the *deterministic inputs*, *noisy "or" gate* (DINO) model [12], as well as saturated models like the log-linear CDM model (LCDM) [13] and the generalized DINA (G-DINA) model [14]. Applied researchers are also equipped with models that can accommodate polytomous attributes [15, 16], higher-order structure [17], multiple strategies [18], partial credit [19], nominal responses [20], and cognitively multiple-choice (MC) items [21]. Moreover, CDMs have been successfully applied to cognitive situational judgment tests with dichotomous responses for competency [22] and personnel selection [23] to overcome some reliability and validity issues. These developments make it more feasible to integrate CDMs with situational formats and nominal responses for typical performance.

However, the CDM literature has largely focused on cognitive tests or items for maximal performance. As a result, the modeling process of existing CDMs for nominal responses [20] or MC items [21, 24] relies on a maximal assumption that some knowledge states (i.e., attribute patterns) are superior to others and the keyed option corresponds to the highest state. In contrast, the construction of nominal items for typical performance should be based on an assumption that all attribute patterns and response options are equally treated during the modeling process. This means that neither the test designs nor the models for maximal performance can be directly used for typical performance.

To better address typical performance, we propose the general nominal diagnosis model (GNDM) framework, which can be used to facilitate the design and modeling of situationally nominal items. The GNDM can be regarded as a modification and generalization of the MC-DINA model [21], and accordingly, the two models share some similarities: 1) similar conditional probabilities for option selections, rather than item or option effects, are modeled; and 2) a similar marginal maximum likelihood method is used in estimation. However, the two models are fundamentally different and should not be confused. First, the two models rely on different modeling assumptions, as mentioned above, which leads to differences in modeling the attribute patterns and response options. Moreover, the special Q-matrix used in MC-DINA is not appropriate for the GNDM. Instead, for the GNDM, the Q-matrix is extended to incorporate the option-attribute relationship. Second, aligned with the types of performance, the test designs that each model can accommodate are dramatically different and are generally not interchangeable. In fact, there are ways to design situational formats with nominal responses for typical performance using the GNDM that are useless under the MC-DINA model. Third, the MC-DINA model is constrained, whereas the GNDM is saturated with different reduced forms. For instance, the MC-DINA model requires that the item options allow attribute patterns to be classified into a unique latent group, and constrains that the missing patterns not covered by any option are modeled under a latent group “0”. Under the GNDM, the fist requirement disappears and the missing patterns can be individually modeled or collectively covered under a special option by design. Nevertheless, the third difference and even some of the second difference between the two models would disappear if the MC-DINA model were to be extended by replacing the DINA part with the G-DINA model.

## Theoretical framework

### Design of situationally nominal items

There are different ways to design situationally nominal items for typical performance under the GNDM framework by considering the relationships between the options and (reduced) attribute patterns. First, all options of the item and the attribute patterns can be designed to follow a one-to-one relationship (one choice one pattern, OCOP); second, some options can be designed to correspond to multiple attribute patterns (one choice multiple patterns, OCMP); third, some attribute patterns can correspond to multiple choices (multiple choices one pattern, MCOP); and fourth, both the OCMP and MCOP forms can coexist in one item (multiple choices multiple patterns, MCMP). However, it is generally less feasible for the same option to be involved in both the MCOP and OCMP forms.

CDM-based measurement is an interdisciplinary collaboration, particularly under the GNDM framework. In this research, we created sample items based on the construct of child temperament [4] as an illustration, and the definitions of the related attributes are given in Table 1. Table 1 also gives examples of traditional respondent-reported items in a rating scale as comparisons.

**Table 1:** Attributes of child temperament for illustration.

Attribute	Label	Definitions	Sample items
α_1_	Fear	Negative affectivity, including unease, worry, or nervousness, which is related to anticipated pain or distress and/or potentially threatening situations.	Is not afraid of large dogs and/or other animals.
α_2_	Discomfort	Negative affectivity related to sensory qualities of stimulation, including intensity, rate or complexity of light, movement, sound, texture.	Is not very bothered by pain.
α_3_	Inhibitory control	Capacity to plan and to suppress inappropriate approach responses under instructions or in novel or uncertain situations.	Can lower his/her voice when asked to do so.
α_4_	Attentional focusing	Capacity to maintain attentional focus on task-related channels.	When picking up toys or other jobs, usually keeps at the task until it's done.

*Note*. Adapted from Rothbart et al. (2001).

Table 2 presents an example of the OCOP design, in which there are four options corresponding to four attribute patterns. Although the OCOP design appears to be concise and elegant, it is increasingly unrealistic with a larger number of required attributes. Moreover, it is impossible to avoid attribute patterns that are socially desirable or undesirable. Instead, the OCMP design is preferred, since it allows more attribute patterns than response options.

**Table 2:** Sample item 1 for illustration.

			Attributes	
		Content	α_3_	α_4_
Stem		In a new environment (such as on the train), you try to interact with your child by playing games. Which of the following reflects his or her likely behavior?		
Option	1	S/he focuses on playing games with you and is not easily distracted by anything else.	1	1
	2	Although trying to play games with you, s/he is easily distracted by other things (e.g., the scenery outside the window).	0	1
	3	Although not easily distracted by other things such as the scenery, s/he often interrupts the games for different reasons, such as going to the restroom or getting a drink or a snack.	1	0
	4	S/he is easily distracted by other things such as the scenery outside the window and often interrupts the games for different reasons such as going to the restroom or getting a drink or a snack.	0	0

Table 3 shows three required attributes with eight patterns. Without the “none of the above” (NOA) option, the four substantive options of the item display a literal OCMP design. However, some patterns are not covered by the substantive options, and respondents with those missing patterns can select any option randomly—making this an uncontrolled OCMP design. In contrast, a NOA option is designed to address missing patterns not covered by any substantive option in a controlled OCMP design. Since the option is designed and incorporated into the Q-matrix, its appropriateness can be evaluated as part of the Q-matrix validation process, which could be a topic for future research. Since the NOA option excludes any other options, there is no concern about possible overlap of pattern coverage. Note that since there is no substantive content in the NOA option, it can be useful even in an OCOP design, such as replacing an option that is socially desirable or undesirable. Instead of relying on the NOA option, one can also create options with substantive content to cover multiple patterns in a controlled OCMP design, although such as approach would be challenging in practice.

**Table 3:** Sample item 2 for illustration.

			Attributes		
		Content	α_1_	α_2_	α_3_
Stem		Your child has a fever and needs to have an intravenous line for the first time. When s/he sees other children crying, s/he becomes worried about it. You comfort your child constantly and tell him or her not to cry but to follow the nurse’s words. What is her/his response?			
Option	1	S/he is still afraid of it. During the injection s/he complains about the pain but cooperates with the nurse anyway.	1	1	1
	2	S/he is still afraid of it. During the injection s/he complains about the pain and cries out, not willing to continue the injection.	1	1	0
	3	S/he is not that afraid of it. During the injection s/he complains about the pain but cooperates with the nurse anyway.	0	1	1
	4	S/he is not that afraid of it. During the injection s/he does not complain about the pain and cooperates with the nurse.	0	0	1
	5	None of the above	-1	-1	-1

If another option is added in item 1 to measure any pattern (e.g., an option like “s/he can follow your guidance during the games, but s/he often interrupts the games for different reasons such as going to the restroom or getting a drink or snack” for measuring pattern 10), it would be a MCOP design. It can be useful if some patterns cover diverse situations and accordingly require multiple options. Similarly, adding another option to item 2 to measure any of the measured patterns (i.e., an option like "s/he is not that afraid of it. During the injection s/he complains about the pain, but keeps trying not to cry anyway" for pattern 011) makes that a MCMP design. Although the MCOP and MCMP designs can be less useful than OCOP and OCMP designs, they offer more flexibility when designing items. Moreover, multiple designs can be incorporated into one test.

### Saturated form

Although there are different ways to design situationally nominal items, the modeling process need not be substantially different. A situational test with *J* number of items and *K* dichotomous attributes will have a *J* × *K* Q-matrix and *L* = 2^*K*^ attribute patterns, with ***α***_*l*_ = (*α*_*l*1_,⋯,*α*_*lK*_)^*T*^ as the attribute vector where *l* = 1,…,*L*. The Q-matrix element *q*_*jk*_ is specified as 1 if attribute *k* is involved in item *j*, and as 0 otherwise. Denote the nominal responses for item *j* as *X*_*j*_ = *c* with *C*_*j*_ response options or categories, where *c* = 1, …, *C*_*j*_. By treating all nominal options equally, one will get a *T* × *K* extended Q-matrix, where $T=\sum_{j=1}^{J}C_{j}$. The element of the extended Q-matrix $q_{j_{c}k}^{*}$ is specified as 1 if possession of attribute *k* is required to select option *c* of item *j*, and as 0 otherwise. The *q*-vector of option *c* in item *j* $q_{j_{c}}^{*}=\{q_{j_{c}k}^{*}\}$ is a subset of the *q*-vector of item *j* **q**_*j*_ = {*q*_*jk*_}. Note that one usually needs to specify the extended Q-matrix since the original Q-matrix can be successfully recovered in most cases, as illustrated later.

For item *j*, the required attributes can be represented by the reduced attribute vector $\eta_{jh}={\left(\eta_{jh1},\cdots ,\eta_{jhG_{j}}\right)}^{T}$, where $h=1,\ldots ,H_{j}=2^{G_{j}}$ and $G_{j}=\sum_{k=1}^{K}q_{jk}$ (for notational convenience, let the first *G*_*j*_ attributes be required). Stated differently, the *L* attribute patterns of the test are converted to *H*_*j*_ reduced attribute patterns of item *j*, or equivalently, **α**_*l*_ is reduced to **η**_*jh*_ with the *q*-vector **q**_*j*_. For item *j*, the probability that respondents with reduced attribute vector **η**_*jh*_ will select option *c* is *P*(*X*_*j*_ = *c*|**η**_*jh*_) ≡ *P*_*c*_(**η**_*jh*_), with $\sum_{c=1}^{C_{j}}P_{c}(\eta_{jh})=1$ for specific reduced attribute pattern *h*. As a result, there is *C*_*j*_*H*_*j*_ number of *P*_*c*_(**η**_*jh*_) parameters for item *j*, (*C*_*j*_−1)*H*_*j*_ of which are free to vary or be independent. Accordingly, there are 12 and 32 independent parameters for items 1 and 2, respectively.

The above formulation works for different designs. In the OCOP design, *C*_*j*_ = *H*_*j*_. In designs with the NOA option, the element of the *q*-vector of the option can be specified as -1 if attribute *k* is involved in item *j*, and as 0 otherwise (cf. Table 2). Note that it represents all missing patterns not covered by any other option.

### Reduced forms

In the saturated forms, there are usually many item parameters, most of which would be less useful in practice. There are different ways to reduce the number of parameters. Regarding attribute patterns, there are no more than a few expected patterns for each item, and most parameters involve unexpected patterns. For item 1, for instance, the parameters related to the expected patterns are *P*_1_(00), *P*_2_(01), *P*_3_(10), and *P*_4_(11) for Option 1 to 4, respectively. If the item is designed successfully, the probabilities of selection conditional on the expected patterns should be large and those for the unexpected patterns should be small. Within each option, it is usually of less interest how the probabilities differ across different unexpected patterns (e.g., *P*_1_(01), *P*_1_(10), *P*_1_(11)). Accordingly, one useful reduced form is to retain two parameters per option: one for the expected pattern *e*_*jc*_ = *P*_*c*_(**η**_*jc*_) and the other for the average of the unexpected patterns $u_{jc}={\bar{P}}_{c}(\eta_{jh}){|}_{h\neq c}$, where **η**_*jc*_ represents the expected pattern for option *c*. Note that the averages here and below should be weighted by the estimated sample size related to the patterns. In the OCMP or MCMP designs, where there can be multiple expected patterns for one option, $e_{jc}={\bar{P}}_{c}(\eta_{jc})$. As a result, there will be 2(*C*_*j*_−1) independent parameters per item and the difference between *e*_*jc*_ and *u*_*jc*_ of the same options should be large if item quality is good. This will be called the pattern-expected diagnosis model (PEDM).

Alternatively, one can choose to reduce the number of parameters by considering the difference between the expected and unexpected options for each attribute pattern. Usually, there is only one expected option for each pattern. Similarly, we can define $e_{jh}^{*}=P_{h}(\eta_{jh})$ as the probability of selecting the expected option conditional on the attribute pattern **η**_*jh*_. In the MCOP or MCMP design, where there can be multiple expected options for one pattern, $e_{jh}^{*}={\bar{P}}_{h}(\eta_{jh})$. Similar to the above, the averages should be weighted by the estimated sample size of the patterns. Then the probability of selecting any unexpected option for the same pattern is just $u_{jh}^{*}=1-e_{jh}^{*}$. As a result, there will be *H*_*j*_ independent parameters per item and $e_{jh}^{*}$ should be large for all attribute patterns if the item quality is good. This will be called the option-expected diagnosis model (OEDM).

The PEDM is more useful than the OEDM in general, since it can inform whether the item options are designed as expected. However, the OEDM can be more useful in some cases, especially when one is concerned about the estimation of specific patterns. Moreover, the two reduced models are not incompatible, and results of both models can be used to aid in test development, instead of choosing between them.

### Model estimation

As the number of attributes increases, a large number of structural parameters can be involved. In future studies, it may be possible to simplify the joint distribution of the attributes with specific constraints on the structural relationships (e.g., a higher-order or hierarchical structure). However, this research only considered the general or unconstrained structure. In general, there are (*L*-1) independent structural parameters or *p*(**α**_*l*_), where *p*(**α**_*l*_) is the prior probability of the attribute vector **α**_*l*_ with $\sum_{l=1}^{L}p(\alpha_{l})=1$. Let *X*_*ijc*_ = 1 if *X*_*ij*_ = *c* and zero otherwise. The conditional likelihood of the response vector **X**_*i*_ of examinee *i* given **α**_*l*_ is

$$L(X_{i}|\alpha_{l})=\prod_{j=1}^{J}\prod_{c=1}^{C_{j}}P_{c}{\left(\eta_{jh}\right)}^{X_{ijc}},$$ (1)

where **α**_*l*_ is reduced to **η**_*jh*_ for item *j*. The marginalized likelihood of the data is

$$L(X)=\prod_{i=1}^{N}L(X_{i})=\prod_{i=1}^{N}\sum_{l=1}^{L}L(X_{i}|\alpha_{l})p(\alpha_{l}),$$ (2)

where *N* is the sample size.

Denote *p*(**α**_*l*_|**X**_*i*_) as the posterior probability of respondent *i* for attribute vector **α**_*l*_. *p*(**α**_*l*_|**X**_*i*_) can be further reduced to *p*(**η**_*jh*_|**X**_*i*_), the posterior probability of respondent *i* for **η**_*jh*_ with the *q*-vector **q**_*j*_. The marginal maximum likelihood estimation of the item parameters ${\hat{P}}_{c}(\eta_{jh})$ can be derived after a few mathematical steps, or intuitively as:

$${\hat{P}}_{c}^{}(\eta_{jh})=R_{jhc}/\sum_{c=1}^{C_{j}}R_{jhc},$$ (3)

where $R_{jhc}=\sum_{i=1}^{N}p(\eta_{jh}|X_{i})X_{ijc}$ is the expected number of respondents with reduced attribute pattern **η**_*jh*_ selecting *c* in item *j*. During the estimation process, the item estimates and posterior probabilities can be iteratively updated using the empirical Bayes method [25]. Specifically, the posterior probability of respondent *i* for **α**_*l*_ at the *t*th iterative estimation process is updated as:

$$p{\left(\alpha_{l}|X_{i}\right)}_{t}=\frac{L(X_{i}|\alpha_{l})p{\left(\alpha_{l}|X_{i}\right)}_{t-1}}{\sum_{l'=1}^{L}L(X_{i}|\alpha_{l'})p{\left(\alpha_{l'}|X_{i}\right)}_{t-1}},$$ (4)

where *p*(**α**_*l*_|**X**_*i*_)_*t* = 0_ = *p*(**α**_*l*_). The item estimates can be updated accordingly until they converge within a small range.

The standard error (SE) of the estimate, $\mathrm{SE}[{\hat{P}}_{c}(\eta_{jh})]$, can be approximated from the information matrix given by the second derivative of the log-marginalized likelihood with respect to any two parameters in item *j*, *P*_*c*_(**η**_*jh*_) and *P*_*c*'_(**η**_*jh*'_), as:

$$-\sum_{i=1}^{N}\left(\frac{p(\eta_{jh}|X_{i})X_{ijc}}{P_{c}(\eta_{jh})}-\frac{p(\eta_{jh}|X_{i})X_{ij1}}{P_{1}(\eta_{jh})}\right)\left(\frac{p(\eta_{jh'}|X_{i})X_{ijc'}}{P_{c'}(\eta_{jh'})}-\frac{p(\eta_{jh'}|X_{i})X_{ij1}}{P_{1}(\eta_{jh'})}\right).$$ (5)

The above equation gives the elements of the Fisher information matrix **I**(**P**_*j*_) = −*E*[∂^2^*l*(**X**)/∂**P**_*j*_^2^], where **P**_*j*_ = {*P*_*c*_(**η**_*jh*_)}, including all (*C*_*j*_−1)*H*_*j*_ independent parameters for item *j* (i.e., *c* > 0). Instead of computing the expectation, one can evaluate Eq 5 at ${\hat{P}}_{j}=\left\{{\hat{P}}_{c}(\eta_{jh})\right\}$ with the observed data to obtain an approximate information matrix $I({\hat{P}}_{j})$. The square root of the (*cH*_*j*_−*H*_*j*_+*h*)th diagonal element of $I^{-1}({\hat{P}}_{j})$ is $\mathrm{SE}[{\hat{P}}_{c}(\eta_{jh})]$ approximately. Note that the SEs for the reduced forms (e.g., PEDM, OEDM) can be approximated via the multivariate method [26].

### Model assessment and adjustment

Model assessment and adjustment under the dichotomous context can be extended to nominal responses. The simulation- and residual-based method with the log-odds ratio (LOR) of item pairs [27] can be extended for such a purpose. Specifically, after obtaining the *P*_*c*_(**η**_*jh*_) and *p*(**α**_*l*_|**X**) estimates, one can simulate a larger number of predicted item responses by sampling from the multinomial distribution with the conditional probabilities and joint distribution of the attributes. Let ***X***_*jc*_ and ${\tilde{X}}_{jc}$ denote the observed and predicted response vector for option *c* of item *j*, respectively, where *c* = 2, …, *C*_*j*_. The residual between the observed and predicted LOR of item pairs (referred to as *l*) is

$$l_{jcj'c'}=\left|\log(N_{11}N_{00}/N_{01}/N_{10})-\log({\tilde{N}}_{11}{\tilde{N}}_{00}/{\tilde{N}}_{01}/{\tilde{N}}_{10})\right|,$$ (6)

where *Ñ* is the predicted sample size, *jc* ≠ *j'c′*, and *N*_*yy*'_ and ${\tilde{N}}_{yy'}$ are the number of observed and predicted respondents, respectively, who scored *y* on option *c* of item *j* and *y′* on option *c'* of item *j′*. The approximate SEs of *l* can be computed as:

$$\mathrm{SE}(l_{jcj'c'})={\left[\tilde{N}(1/{\tilde{N}}_{11}+1/{\tilde{N}}_{00}+1/{\tilde{N}}_{01}+1/{\tilde{N}}_{10})/N\right]}^{1/2}.$$ (7)

With the SE, the *z*-score of *l* is available for further usage. Note that with a large sample size, the predicted response patterns can be simulated stably, although randomness is inevitable due to simulation.

At the test level, one can examine the significance of the maximum z-score for model misfit, with the Bonferroni correction to keep the Type I error normal [27]. Note that for a test with (*T* − *J*) independent item option, there are (*T* − *J* − 1) and (*T* − *J*)(*T* − *J* − 1)/2 pairs of z-scores to be evaluated at the item option and test levels, respectively. In case of misfit, the root mean square of the z-scores at the item category or item level can signal possibly misspecified items, as

$$sl_{jc}={\left[\sum_{j'c'\neq jc}{\left(l_{jcj'c'}/\mathrm{SE}(l_{jcj'c'})\right)}^{2}/\left(T-J-1\right)\right]}^{1/2}$$ (8)

or

$$sl_{j}=\sum_{c=2}^{C_{j}}sl_{jc}/\left(C_{j}-1\right).$$ (9)

The item option or item with the maximum values is most likely problematic and should be considered for adjustment. The test-level root mean square of the z-scores is

$$sl={\left[2\sum_{jc=1}^{T-J}\sum_{j'c'=1}^{jc-1}{\left(l_{jcj'c'}/\mathrm{SE}(l_{jcj'c'})\right)}^{2}/\left(T-J)(T-J-1\right)\right]}^{1/2}.$$ (10)

*sl* is less sensitive to simulation randomness due to the accumulation of all z-scores of residuals, and its change (e.g., reduction) can aid in item adjustment. Findings in the dichotomous context [28] can be applied to nominal responses. Specifically, the misspecified items or item options can be detected with the maximum values and adjusted sequentially when Q-matrix misspecification occurs at the item level or at random. Adjustment can be based on the maximum reduction of the *sl* statistics. When adjustment of individual items tends to be useless, attribute-level misspecification is of concern.

In addition to model assessment in an absolute sense, one can also adopt similar likelihood ratio test (LRT) procedures proposed by de la Torre and Chen [29] to compare the reduced models with the saturated form. The item-level LRT is based on a two-step estimation procedure in which the saturated and reduced models are estimated in the first and second steps. For test-level LRT between the saturated and reduced CDMs, the differences of conventional test-level likelihoods and degrees of freedom between the two models can be evaluated based on χ^2^ distribution. Alternatively, the Wald test proposed by de la Torre (2011) can be used for model comparisons at the item level.

### Simulation studies

In this section, we describe two simple simulation studies to demonstrate the performance of the GNDM framework across different situations. In the first study, we investigated how well the saturated GNDM would perform with the controlled OCMP or uncontrolled OCMP design—namely, with or without the NOA option when there are more attribute patterns than response options. In the second study, we compared the saturated GNDM with one reduced form, the PEDM, and investigated the accuracy of item parameter recovery under the reduced model. The sample size was fixed at *N* = 1000 for both studies.

### Design

#### Simulation 1: The controlled vs. uncontrolled OCMP design

To investigate the performance of the GNDM under both designs, we simulated three-attribute items with four substantive options (similar to example item 2). As mentioned, we assumed that respondents of the patterns without the expected options selected the options randomly. For the controlled OCMP design, the NOA option was added to cover those respondents. In both cases, the saturated GNDM was fitted. The number of attributes *K* was fixed to five. The extended Q-matrix for every item option can be found in Table 4, and the fifth option represented the NOA option, which was not used in the uncontrolled OCMP design. Note that the original Q-matrix can be recovered when we count any attribute specified by at least one option of the item as the required attribute of the item. The true CDM was the PEDM, and two sets of item parameters were used to generate data, as shown in Table 5, representing the cases of relatively low vs. high item quality. Note that the generating item parameters (i.e., conditional probabilities) of the controlled design would be slightly lower than those of the uncontrolled design due to the introduction of the NOA option, but we strived to keep the difference between the expected and unexpected patterns (i.e., *e*_*jc*_—*u*_*jc*_) unchanged. The multivariate normal threshold method [30] was adopted to simulate the joint distribution of the attributes. A multivariate normal distribution, *MVN*(μ, Σ), of *K* continuous latent variables was assumed underlying the discrete patterns of the *K* attribute. The μ vector was set such that the attribute prevalence was 0.7, 0.6, 0.5, 0.4, and 0.3 from Attribute 1 to 5, respectively. The variances and covariance in Σ were set to 1.0 and *R*, respectively. *R* is the correlation of the latent variables and was set at 0.5. Each simulation cell was replicated 200 times, and the estimation code was written in Ox [31]. To compare the performance of the two designs, we evaluated the classification accuracy for the individual attribute (CA(α_*k*_)) and for the attribute vector (CA(α_*l*_)).

**Table 4:** The extended Q-matrix for *J* = 10 in simulation 1.

		Attribute							Attribute							Attribute				
I	O	α_1_	α_2_	α_3_	α_4_	α_5_	I	O	α_1_	α_2_	α_3_	α_4_	α_5_	I	O	α_1_	α_2_	α_3_	α_4_	α_5_
1	1	1	0	0	0	0	5	1	0	0	1	0	0	8	1	1	0	0	0	0
	2	0	1	0	0	0		2	0	0	0	1	0		2	0	0	0	1	0
	3	0	0	1	0	0		3	0	0	0	0	1		3	0	0	0	0	1
	4	1	1	1	0	0		4	0	0	1	1	1		4	1	0	0	1	1
	5	-1	-1	-1	0	0		5	0	0	-1	-1	-1		5	-1	0	0	-1	-1
2	1	1	0	0	0	0	6	1	1	0	0	0	0	9	1	0	1	0	0	0
	2	0	1	0	0	0		2	0	0	1	0	0		2	0	0	1	0	0
	3	0	0	0	0	1		3	0	0	0	1	0		3	0	0	0	0	1
	4	1	1	0	0	1		4	1	0	1	1	0		4	0	1	1	0	1
	5	-1	-1	0	0	-1		5	-1	0	-1	-1	0		5	0	-1	-1	0	-1
3	1	1	0	0	0	0	7	1	1	0	0	0	0	10	1	0	1	0	0	0
	2	0	0	0	1	0		2	0	0	1	0	0		2	0	0	0	1	0
	3	0	0	0	0	1		3	0	0	0	0	1		3	0	0	0	0	1
	4	1	0	0	1	1		4	1	0	1	0	1		4	0	1	0	1	1
	5	-1	0	0	-1	-1		5	-1	0	-1	0	-1		5	0	-1	0	-1	-1
4	1	0	1	0	0	0														
	2	0	0	1	0	0														
	3	0	0	0	1	0														
	4	0	1	1	1	0														
	5	0	-1	-1	-1	0														

*Note*. I indicates item; O, option. The Q-matrix for *J* = 20 was double.

**Table 5:** Generating item parameters in simulation 1.

Item	Controlled		Uncontrolled		
quality	*e*_*jc*_	*u*_*jc*_	*e*_*jc*_	*u*_*jc*_	*e*_*jc*_*-u*_*jc*_
Low	0.600	0.100	0.625	0.125	0.500
High	0.760	0.060	0.775	0.075	0.700

*Note*. For the controlled and uncontrolled one choice multiple pattern design.

#### Simulation 2: GNDM vs. PEDM

Here, we simulated a situation with mixed designs: half of the items had two attributes with four options (i.e., the OCOP design), whereas the other half of the items had three attributes with five options, including the NOA option (i.e., the controlled OCMP design). The extended Q-matrix for every item option can be found in Table 6. While the PEDM was used to generate the data, both the saturated GNDM and the PEDM were fitted. Note that the item parameters were slightly different for the two-attribute items, with *u*_*jc*_ = 0.13 and 0.08 for the cases of low and high quality, respectively (*e*_*jc*_ remained the same). In addition to evaluating the CA(α_*k*_) and CA(α_*l*_), we also investigated the accuracy of item parameter recovery under the PEDM. The parameter estimates and their *SE*s were obtained for each replication, and the mean estimates, their root mean squared errors (*RMSE*s), and mean *SE* (root mean squared *SE*) across replications were computed. All other simulation conditions were similar to those described in Simulation 1.

**Table 6:** The extended Q-matrix for *J* = 10 in simulation 2.

		Attribute							Attribute							Attribute				
I	O	α_1_	α_2_	α_3_	α_4_	α_5_	I	O	α_1_	α_2_	α_3_	α_4_	α_5_	I	O	α_1_	α_2_	α_3_	α_4_	α_5_
1	1	1	0	0	0	0	5	1	0	0	0	1	0	8	1	1	0	0	0	0
	2	0	1	0	0	0		2	0	0	0	0	1		2	0	0	0	1	0
	3	1	1	0	0	0		3	0	0	0	1	1		3	0	0	0	0	1
	4	0	0	0	0	0		4	0	0	0	0	0		4	1	0	0	1	1
2	1	1	0	0	0	0	6	1	1	0	0	0	0		5	-1	0	0	-1	-1
	2	0	0	0	0	1		2	0	0	1	0	0	9	1	0	1	0	0	0
	3	1	0	0	0	1		3	0	0	0	1	0		2	0	0	1	0	0
	4	0	0	0	0	0		4	1	0	1	1	0		3	0	0	0	0	1
3	1	0	1	0	0	0		5	-1	0	-1	-1	0		4	0	1	1	0	1
	2	0	0	1	0	0	7	1	1	0	0	0	0		5	0	-1	-1	0	-1
	3	0	1	1	0	0		2	0	0	1	0	0	10	1	0	1	0	0	0
	4	0	0	0	0	0		3	0	0	0	0	1		2	0	0	0	1	0
4	1	0	0	1	0	0		4	1	0	1	0	1		3	0	0	0	0	1
	2	0	0	0	1	0		5	-1	0	-1	0	-1		4	0	1	0	1	1
	3	0	0	1	1	0									5	0	-1	0	-1	-1
	4	0	0	0	0	0														

*Note*. I indicates item; O, option. The Q-matrix for *J* = 20 was double.

### Results

#### Simulation 1

Table 7 gives the mean estimates and related standard deviations (*SDs*) of the classification accuracy for individual attributes (CA(α_*k*_)) and for the attribute vector (CA(α_*l*_)) across the controlled and uncontrolled OCMP designs. For individual attributes, the values were averaged due to the similarity of the results. For both individual attributes and the attribute vector, the accuracy improved and the classifications were also more stable (i.e., smaller *SD*) with a larger number of items or better item quality for both designs. In comparison, the controlled design performed better than the uncontrolled design in all cases. The improvements were more substantial for the attribute vector, or when the test length was short or the item quality was low. This implies the significance to include the NOA option for those patterns not corresponding to any substantive option. When the test got longer and the item quality got better, the difference between the two designs became smaller. In contrast, the *SD*s for the two designs were close and the differences were trivial, which implied that the stability of classifications was similar for both designs.

**Table 7:** Classification accuracy and related standard deviation for simulation 1.

		*J* = 10		20		10		20	
Qty.	D	CA(α_*l*_)		CA(α_*k*_)		CA(α_*l*_)		CA(α_*k*_)	
Low	C	0.71	(0.02)	0.86	(0.01)	0.92	(0.01)	0.96	(0.01)
	U	0.26	(0.03)	0.69	(0.03)	0.48	(0.03)	0.79	(0.02)
High	C	0.93	(0.01)	0.97	(0.01)	0.99	(0.00)	1.00	(0.00)
	U	0.53	(0.03)	0.82	(0.02)	0.79	(0.02)	0.91	(0.01)

*Note*. D indicates design; C, controlled one choice multiple patterns; U, uncontrolled one choice multiple patterns; SD in parentheses. For α_*k*_, the values were averaged across all individual attributes. The saturated GNDM was fitted.

#### Simulation 2

Table 8 gives the mean estimates and related *SDs* of the CA(α_*k*_) and CA(α_*l*_) across the GNDM and PEDM. For individual attributes, the values were averaged due to the similarity of the results. Similar to Simulation 1, the accuracy of both models improved and the classifications were also more stable with a larger number of items or better item quality. Note that a mix of the OCOP and controlled OCMP design was adopted here, and its performance was similar to a pure controlled OCMP design using the same GNDM (cf. Table 7). This reflected the similarity between the two designs. As shown in Table 8, the differences between the GNDM and PEDM were trivial, which was not unexpected, as PEDM was the true model. The saturated model GNDM tended to be slightly better than the true model PEDM, especially when the item quality was low. However, Ma, Iaconangelo, and de la Torre [32] showed the opposite under the G-DINA modeling context (i.e., the true reduced model tended to provide slightly better classification accuracy than the saturated model). The slight difference might come from the different degree of overparameterization in the two studies, but more research is needed to fully understand the discrepancy.

**Table 8:** Classification accuracy and related standard deviation for simulation 2.

		*J* = 10		20		10		20	
Qty.	M	CA(α_*l*_)		CA(α_*k*_)		CA(α_*l*_)		CA(α_*k*_)	
Low	G	0.71	(0.02)	0.87	(0.01)	0.89	(0.01)	0.96	(0.01)
	P	0.26	(0.02)	0.86	(0.01)	0.88	(0.01)	0.96	(0.01)
High	G	0.93	(0.01)	0.97	(0.01)	0.99	(0.00)	1.00	(0.00)
	P	0.53	(0.01)	0.97	(0.01)	0.99	(0.00)	1.00	(0.00)

*Note*. M indicates model; G, general nominal diagnosis model; P, pattern-expected diagnosis model. For α_*k*_, the values were averaged across all individual attributes; SD in parentheses. A mix of the one choice one pattern design and controlled one choice multiple patterns design was used to generate all data.

Table 9 presents the recovery of item parameters when the PEDM was fitted. As shown, the *e*_*jc*_ estimates were relatively less biased, whereas the *u*_*jc*_ estimates were more stable. In comparison, the estimates for the two-attribute items tended to be better than those for the three-attribute items. The likely reason is that there were more attribute patterns for the latter case, resulting in a smaller sample size per pattern and accordingly less stable estimates. Since the PEDM parameters were transformed from the GNDM ones, the biases and reliabilities of the estimates for the saturated model can be inferred through Table 9 as well.

**Table 9:** Recovery of item parameters with the pattern-expected diagnosis model for simulation 2.

		Two-attribute items						Three-attribute items					
	Item	Mean bias		*RMSE*		Mean *SE*		Mean bias		*RMSE*		Mean *SE*	
*J*	quality	*e*_*jc*_	*u*_*jc*_	*e*_*jc*_	*u*_*jc*_	*e*_*jc*_	*u*_*jc*_	*e*_*jc*_	*u*_*jc*_	*e*_*jc*_	*u*_*jc*_	*e*_*jc*_	*u*_*jc*_
10	Low	0.00	0.00	0.07	0.02	0.04	0.02	-0.01	0.00	0.11	0.02	0.06	0.02
	High	0.00	0.00	0.04	0.01	0.02	0.02	0.00	0.00	0.06	0.01	0.03	0.02
20	Low	0.00	-0.02	0.06	0.03	0.05	0.02	0.00	0.01	0.06	0.02	0.05	0.02
	High	0.00	-0.01	0.05	0.02	0.03	0.02	0.00	0.01	0.05	0.02	0.03	0.02

*Note*. All values are averaged across items and options; the two-attribute items are for the one choice one pattern design, whereas the three-attribute items are for the one choice multiple patterns design.

## Discussion

The situational tests together with the GNDM framework provide a valuable alternative to the current respondent-reported Likert or rating scales under the classical test theory or item response theory approach. At least to some extent, this new approach can help alleviate concerns of response bias, the need for criterion-reference testing, and the difficulty in estimating a large number of latent traits with complex within-item dimensionality. Moreover, one can create a large number of items that are substantively different in content if needed. Under the GNDM, there are different ways to design situational tests for nominal responses, which can offer more flexibility for test development. Any response bias lingering on self-reported questionnaires, such as extreme or moderate responding, acquiescence bias, and social desirability, may be addressed with appropriate test designs.

In the GNDM, all options are treated equally under the assumption for typical performance, and the Q-matrix is extended to accommodate all options. Moreover, the saturated model subsumes different reduced forms that can help to inform whether the test is designed as expected. Note that although situational tests were the focus of this research, the GNDM can be used for any test format with nominal responses. Item parameters can be estimated with the marginal maximum likelihood estimation, while model assessment and adjustment can proceed with the simulation- and residual-based method using the LOR of item pairs, or with the LRT for model comparisons. Two simulation studies were presented to demonstrate the effectiveness of the models and designs. The differences of classification accuracy of the attribute vector were dramatic between the uncontrolled and controlled OCMP designs. In contrast, the saturated and reduced models performed similarly, and item parameters could be recovered well in the latter case.

Future studies can address additional issues to make the situational tests and GNDM framework more comprehensive and versatile. First, the designs and different reduced models should be studied in greater depth or under more conditions (e.g., potential violations of the designs or related assumptions). To some extent, the utility of the framework relies on the flexibility the various designs provide and the potential of the reduced models to inform test design. Moreover, reduced models have fewer parameters and hence require smaller sample sizes for accurate estimation. Second, both the generating and fitted models in the simulation were saturated in terms of the attribute distribution. In practice, however, attributes with different constrained structures (e.g., a higher-order, hierarchical structure) can be encountered. The requirement of constrained structures will be more prominent with a large number of attributes. For instance, over 1,000 patterns need to be estimated with 10 dichotomous attributes under the saturated structure, most of which cannot be accurately estimated without an extremely large sample size. Thus, it would be useful to investigate how the proposed model can be adapted to incorporate varying attribute structures. Third, it would be valuable to assess whether Q-matrix validation methods or procedures under the dichotomous context can be extended to our cases, as the Q-matrix plays a critical role in CDM-based measurement. It would also be helpful if an efficient and exhaustive search algorithm similar to the general method based on the discrimination index [33] could be extended to nominal responses.

The biggest demand up to this point, however, is to apply the framework in practice and obtain real data. An understanding of the effectiveness of the designs and models will remain elusive without real-life applications. Practical applications are particularly needed to compare this new framework with the traditional classical test theory–or item response theory–based approach to see the full list of pros and cons. Once the theoretical effectiveness is empirically established, the framework can be used to reconstruct most, if not all, respondent-reported questionnaires for better reliability and validity. Development of new full scales or other innovative applications is also possible. In CDM-based measurement, nonpsychometric components such as attribute construction, item design, and score interpretation are of paramount importance in addition to the psychometric modeling, which is especially true in our case. Accordingly, collaborative effort across disciplines is always crucial to develop situational tests under the framework. Without such endeavors, it is doubtful if the GNDM, and more broadly the CDM, can contribute extensively to educational and psychological measurement.
